# Venous Cannulation Site Stenosis and Risk of Recurrence: A New Hypothesis

**DOI:** 10.7759/cureus.92950

**Published:** 2025-09-22

**Authors:** Raquel Pinto, André Ferreira, Joaquim Milheiro, Catarina Veiga, Adriana Dias, Andreia Silva, Sérgio Lemos, Pedro Bizarro

**Affiliations:** 1 Nephrology, Unidade Local de Saúde de Viseu Dão-Lafões, Viseu, PRT; 2 Nephrology, Casa de Saúde da Boavista, Porto, PRT

**Keywords:** arteriovenous fistula, cannulation injury, hemodialysis access, needle cannulation, needle injury, vascular access, venous stenosis

## Abstract

Background: Maintenance of vascular access (VA) patency in hemodialysis (HD) can be challenging and may require multiple endovascular interventions. Changes in local hemodynamics and vessel wall injury caused by needle cannulation are related to a higher risk of neointimal hyperplasia and stenotic lesions. We proposed to analyze the incidence of venous cannulation site stenosis (VCSS) requiring angioplasty and the need for reintervention.

Methods: This is a retrospective cohort single-center study conducted on 431 HD patients with arteriovenous fistulas (AVFs) subjected to stenosis correction in 2022. Descriptive and comparative statistical analyses were performed.

Results: A total of 544 procedures on 438 AVFs were analyzed. The median age was 72 (62-80) years. The most commonly intervened autologous VA was the brachiocephalic (53.4%; n = 234), and of all AVFs, 19.6% (n = 86) were intervened on more than once. The most common stenosis locations were in not otherwise specified (NOS) venous segments (32.4%; n = 176), followed by cephalic arch (30.9%; n = 168) and venous cannulation sites (25.6%; n = 139). Of the AVFs with VCSS identified on first intervention (n = 98), 35.7% (n = 35) had to be reintervened, of which 71.4% (n = 25) recurred on the same site. The intervention on this kind of stenosis is more likely to occur in proximal AVF (p < 0.001) and has a higher risk of needing reintervention (p = 0.004).

Conclusions: There seems to be a relation between VCSS and risk for restenosis, highlighting the role of needle cannulation techniques (including length of cannulation tunnel) and blood flow at cannulation sites in the development of stenotic lesions.

## Introduction

End-stage renal disease is the terminal stage of chronic kidney disease (CKD), and a large number of patients who reach this phase will opt for renal replacement therapy. Among the available modalities, hemodialysis (HD) is the most prevalent and requires a functioning vascular access (VA) [1,2].

The arteriovenous fistula (AVF) is associated with the best outcomes and is usually the preferred VA, since it is associated with lower infection risk and thrombosis, longer survival, and lower risk of morbidity [2-5]. Nevertheless, the Kidney Disease Outcomes Quality Initiative (KDOQI) Clinical Practice Guideline for Vascular Access considers it reasonable to decide on the choice of VA in accordance with the ESKD (End-Stage Kidney Disease) Life-Plan [6]. The preservation of the AVF is of the utmost importance, albeit sometimes challenging and requiring multiple interventions to maintain its patency.

While thrombosis is the predominant cause of fistula loss, a stenosis usually precedes it, making its prevention and early detection fundamental. Stenosis of the autologous VA occurs from neointimal hyperplasia and vascular remodeling (most often in the venous segment), which is influenced by upstream and downstream events [4,7,8]. Upstream events are related to direct vessel wall injury following needle punctures and hemodynamic shear stress, while downstream events are the result of vascular wall response to injury, and comprise an inflammatory response cascade and cell proliferation. This vascular remodeling may develop de novo after AVF construction or be present prior to surgery, in close relation with patient age and comorbidities such as hypertension, cardiovascular disease, peripheral artery disease, and particularly diabetes [3,4,9]. Complications of advanced CKD itself and long-standing uremia also seem to play a role in progressive venous neointimal hyperplasia prior to AVF construction [9].

Physical examination of the AVF is essential to help identify VA complications promptly, which, together with indirect parameters (such as Kt/V, blood flow rates (Qb), and dynamic pressure), should be sufficient to infer the presence of stenosis and allow us to identify its most probable location [3,6]. The crescent use and availability of point-of-care ultrasound should further increase the accuracy of detecting VA problems and allow for its swifter correction [10]. Earlier detection of VA flow dysfunction allows us to correct stenosis earlier, minimize the risk of thrombosis, and prevent access failure. Prompt intervention helps reduce overall morbidity and mortality, which are associated with multiple access interventions and use of central venous catheters [6].

The most common sites of VA stenoses tend to be in areas of more turbulent blood flow, such as the peri-anastomotic region or at the graft-vein anastomosis (in arteriovenous grafts (AVGs)). However, it is also well-known that there is a high incidence of stenosis located near sites of venous needle punctures, as has also been encountered in the authors’ experience. This can be explained by incorrect cannulation techniques, disturbance of normal laminar blood flow during treatments owing to high pump speeds, and the use of metal needles, which seem to be associated with greater blood jet streams and higher mean Doppler velocities at cannulation sites [5,11-14], increasing the likelihood of neointimal proliferation.

The aim of the present study is to assess the incidence of angioplasty-requiring stenosis in venous cannulation sites, henceforth referred to as venous cannulation site stenosis (VCSS), and the need for future reintervention. Definitions for each type of stenosis were based on the Spanish Clinical Guidelines on Vascular Access for Haemodialysis [15]. We chose to divide venous segment stenosis into arterial cannulation site stenosis (ACSS), VCSS, and stenosis that occurred along the venous segment in areas other than these two locations (hereafter NOS stenosis).

## Materials and methods

This is an observational, retrospective, cohort, single-center study conducted on a group of Portuguese HD patients with AVF who underwent stenosis correction in 2022, having all procedures being performed by the same interventional nephrologist. The sample was non-randomized and selected by convenience. The inclusion criteria comprised patients with any form of autologous VA stenosis requiring angioplasty. Exclusion criteria included the following: absence of stenosis on angiography, presence of an AVG, prior implantation of stents or other synthetic materials in the VA lumen, and cases with incomplete procedure reports.

Data were collected from procedure reports (all written by the same interventional nephrologist), and included gender and age, type of VA, number of interventions per VA, and types of stenosis intervened. Patient consent was obtained in written form prior to each intervention, in accordance with the institution's ethics protocols. All data were anonymized.

From a total of 666 endovascular procedures, we excluded 122: 42 due to insufficient reported data, 58 referred to stenosis interventions on AVG, 11 were related to stenosis on AVF with portions of synthetic material, and 11 showed no stenosis on angiography. In the end, we considered 544 procedures on AVF, referring to 438 AVF from 431 different patients.

Statistical analysis was done with SPSS Statistics® for Apple macOS®, version 28.0.1.0 (IBM Corp., Armonk, NY). We used the Shapiro-Wilk normality test. For descriptive analysis, we resorted to frequencies, median, interquartile range (IQR), and minimum and maximum, while for inferential analysis, we used Fisher’s exact test and relative risk (RR) estimation. A p-value of <0.05 was considered statistically significant.

## Results

Definitions for each type of stenosis can be found in Table 1. Classifications were based on the Spanish Clinical Guidelines on Vascular Access for Haemodialysis [15]. We chose to divide venous segment stenosis into ACSS, VCSS, and stenosis that occurred along the venous segment in areas other than these two locations (hereafter NOS stenosis).

**Table 1:** Definitions of different types of stenosis according to the Spanish Clinical Guidelines on Vascular Access for Haemodialysis. Copyright/License: This table has been adapted from Spanish Clinical Guidelines on Vascular Access for Haemodialysis [15], which is an open-access article distributed under the terms and conditions of a CC BY-NC-ND 4.0 license. AVF: arteriovenous fistula; VA: vascular access.

Type of stenosis	Definition
Arterial stenosis	Located in the arterial portion of the AVF. Usually due to lesions resulting from progression of underlying arterial disease.
Stenosis of the anastomosis	Located at the anastomosis of the AVF. Most often related to surgical construction of the VA.
Juxta-anastomotic stenosis	Anywhere from immediately after the anastomosis to 5 centimeters proximal to the anastomosis. Associated with hemodynamic factors and vascular endothelium changes to inflammatory response.
Stenosis in the venous segment	Located in areas of needle cannulation. Secondary to mechanic trauma by vessel punctures.
Cephalic arch stenosis	In the cephalic vein segment immediately adjacent to its confluence into the axillary vein. Associated with hemodynamic factors.
Central vein stenosis	From the subclavian vein to the right atria (includes axillary and subclavian veins, brachiocephalic trunk and superior vena cava). Often related to endothelium trauma secondary to prior presence of central venous catheters.

Among our cohort of patients, the majority were male (62.2%; n = 268). The median age was 72 (minimum = 28 and maximum = 97) years. Referring to the AVF observed, more than half were brachiocephalic (53.4%; n = 234), followed by radiocephalic (26.9%; n = 118). The frequency of each type is represented in Table 2.

**Table 2:** Frequency of types of arteriovenous fistulas observed. AVF: arteriovenous fistula; N: number; VA: vascular access; %: percentage.

Type of VA	N	%
Brachiocephalic AVF	234	53.4
Radiocephalic AVF	118	26.9
Brachiobasilic AVF	75	17.1
Brachiomedian AVF	7	1.6
Brachiobrachial AVF	2	0.5
Saphenous loop AVF	2	0.5

Table 3 displays the total number of stenoses encountered (n = 699) on the intervened AVF. It should be noted that many of the VAs had multiple stenoses requiring angioplasty during the same intervention.

**Table 3:** Frequency of stenosis by type. AVF: arteriovenous fistula; ACSS: arterial cannulation site stenosis; N: number; NOS: not otherwise specified; VCSS: venous cannulation site stenosis; %: percentage.

Type of stenosis	N	% of stenosis in all AVFs
Arterial	8	1.5
Anastomosis	56	10.3
Juxta-anastomotic	104	19.1
ACSS	25	4.6
VCSS	139	25.6
NOS venous segment	176	32.4
Cephalic arch	168	30.9
Central vein	23	4.2

Most stenoses were located in NOS venous segments (32.4%; n = 176), followed by stenosis in the cephalic arch (30.9%; n = 168) and VCSS (25.6%; n = 139). Inferential analysis was done to assess the risk of needing stenosis correction in proximal versus distal AVF. Results reveal a much higher risk of cephalic arch stenosis (39.4% vs. 5.2%; RR: 7.59, 95% CI (3.65-15.77), p < 0.001) and VCSS (28.6% vs. 16.3%; RR: 1.76, 95% CI (1.16-2.65), p = 0.004) in proximal AVF than in distal ones. On the contrary, there was a lesser risk for stenosis in the arterial segment (0.2% vs. 5.2%; RR: 0.05, 95% CI (0.01-0.38), p < 0.001), anastomosis (5.9% vs. 23.7%; RR: 0.25, 95% CI (0.15-0.41), p < 0.001), juxta-anastomotic (13.4% vs. 36.3%; RR: 0.37, 95% CI (0.27-0.52), p < 0.001), and ACSS (2.7% vs. 10.4%; RR: 0.26, 95% CI (0.12-0.56), p < 0.001) (Table 4).

**Table 4:** Risk for needing stenosis angioplasty in proximal (vs. distal) AVF. P-values were obtained using Fisher's exact test. ACSS: arterial cannulation site stenosis; AVF: arteriovenous fistula; VCSS: venous cannulation site stenosis.

Type of stenosis	Relative risk	95% CI	P-value
Arterial	0.05	0.01-0.38	<0.001
Anastomosis	0.25	0.15-0.41	<0.001
Juxta-anastomotic	0.37	0.27-0.52	<0.001
ACSS	0.26	0.12-0.56	<0.001
VCSS	1.76	1.16-2.65	0.004
Cephalic arch	7.59	3.65-15.77	<0.001
Central vein	3.47	0.82-14.59	0.830

As for reinterventions, 80.4% (n = 352) of all AVFs were intervened on only once, while 19.6% (n = 86) were intervened on between two and four times during the period of our study. Our results demonstrate VCSS had a higher risk of needing a new intervention vs. not needing it (28.8% vs. 16.3%; RR: 1.77, 95% CI (1.25-2.47), p = 0.002), while stenosis at the anastomosis (8.9% vs. 20.7%; RR: 0.43, 95% CI (0.18-1.01), p = 0.033) had a lower risk. Arterial, cephalic arch, and central vein stenosis also had an RR >1 of requiring reintervention, but did not reach statistical significance (Table 5). It is also worth mentioning that, of the VCSS reinterventions (n = 35), 71.4% (n = 25) had recurred on the same site.

**Table 5:** Risk of needing stenosis reintervention based on the location of primary stenosis. P-values were obtained using Fisher's exact test. ACSS: arterial cannulation site stenosis; VCSS: venous cannulation site stenosis.

Type of stenosis	Relative risk	95% CI	P-value
Arterial	1.29	0.38-4.33	0.657
Anastomosis	0.43	0.18-1.01	0.033
Juxta-anastomotic	0.59	0.35-1.01	0.053
ACSS	0.61	0.21-1.77	0.443
VCSS	1.77	1.25-2.47	0.002
Cephalic arch	1.01	0.70-1.46	1.000
Central vein	1.12	0.51-2.48	0.790

## Discussion

Prevalence of different types of arteriovenous fistulas

The most prevalent AVF in our patients was the brachiocephalic, and one explanation may be due to the older age of our patients, with a median age of 72 years. Although we were not able to gather information about comorbidities and other underlying disorders, hypertension, diabetes mellitus, and cardiovascular disease are incredibly prevalent diseases in the Portuguese population [16]. These are known risk factors for atherosclerosis and peripheral artery disease [17], and although these comorbidities should not limit distal AVF construction, it is true that these comorbid conditions increase the rate of primary AVF failure [2].

Prevalence of different types of stenosis

The type of stenosis most frequently encountered was in NOS venous segments, followed by stenosis in the cephalic vein arch. However, we must highlight the numbers referring to VCSS, our primary outcome, which were present in more than 25% of all AVFs studied, being the third most frequent type of stenosis observed in our sample. Nonetheless, those classified as NOS venous segment stenosis may be underestimating the total number of stenoses associated with venous needle cannulation.

There is already evidence in published literature that supports our findings. First, these sites are frequently punctured and consequently prone to developing stenotic lesions, secondary to direct trauma to the vessel wall, and when needle repositioning is necessary, increasing the risk of back wall damage [11-13]. Secondly, blood-flow disturbances cause changes in wall shear stress (WSS) and activate biochemical cascades, inducing cell proliferation. These are influenced by blood flow at cannulation sites and by the type of needle used. Doppler studies on AVF revealed that a higher Qb (400 ml/min and greater) produces higher mean velocities and unstable jets, which tend to be less turbulent at Qb of 300 ml/min [11,12,14]. Also, metal needles produce distinct blood flow patterns compared to plastic cannulae. The former creates a solid stream toward the vessel wall, whereas the latter allows for the dispersion of blood through the side holes, directing it to the center of the vessel and reducing turbulence [11,12]. In our study, all VAs were cannulated using metal needles, so we could not assess this variable.

Other aspects of cannulation may contribute to VCSS: the angle and direction of venous needle insertion influence the impact of the jet stream, and areas of recirculation seem to encourage deposition of particles, increasing the propensity to stenosis formation. In the computational studies by Fulker et al. [5,14], WSS was lower when the needle was inserted at a shallow angle and placed in the center of the vein, with normal venous flow playing an important role in dissipating the needle jet and minimizing its impact on the vein wall.

Prevalence of different types of stenosis in proximal vs. distal arteriovenous fistulas

We found a statistically significantly higher risk of developing cephalic arch stenosis (p < 0.001) and VCSS (p = 0.004) in proximal fistulas when compared to distal AVF. The former comes as no surprise, as the association between proximal fistulas and a greater incidence of cephalic arch stenosis has been extensively documented [2,18-21]. This higher risk is thought to be influenced by more turbulent flow in the curvature of the cephalic vein, not only due to its anatomical design but also due to external compression by fascia and muscles, which limits the vessel's capability to remodel and dilate in response to increase in flow rates, as is in five of seven cases of a dialysis VA [2,18,21]. On the other hand, the finding of a higher risk for VCSS in proximal fistulas has not been previously reported in the literature, to our knowledge, and we can only propose theories for this finding. The fact that a proximal VA receives greater blood flow than distal ones may justify greater turbulence downstream. Such factors could easily be implicated in an also greater susceptibility to vessel wall damage at these sites, with consequent stenosis development.

Endovascular procedures as a risk factor for restenosis

Endovascular interventions, per se, have a known risk of inducing endothelial injury and increasing the risk of restenosis [7,8,11,12]. In fact, Chang et al. demonstrated that intima and media layers of the AVF that had been submitted to multiple endovascular interventions showed greater cell proliferation activity when compared to AVF with a first stenotic event [7].

As for reinterventions, our results point to a significantly increased risk of needing subsequent procedures in the case of VCSS (p = 0.002). What is more, 71.4% of these occurred on the same site as before. Several factors may contribute to this finding: (1) endothelial damage induced by angioplasty that perpetuates the formation of restenosis; (2) association of the primary lesion with a risk for recurrence; (3) continued exposure to another factor that is the source of the stenotic lesion, such as cannulation technique. While it is known that rope-ladder cannulation is the preferred technique due to its lower rate of complications [1,6,13], area cannulation had unfortunately been the most used practice in the VA observed in our study.

**Figure 1:**
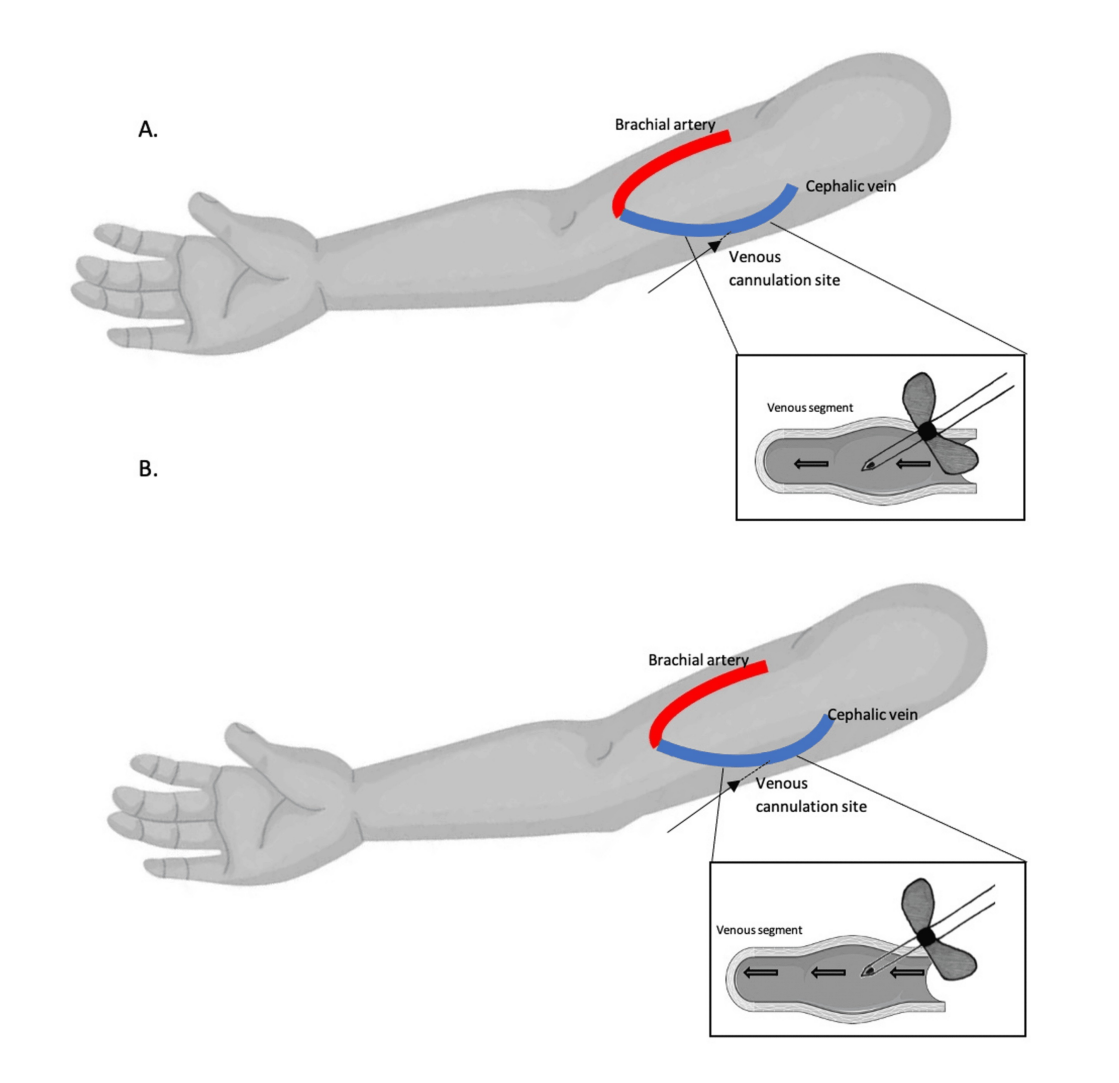
Example of short cannulation tunnels. (A) Short subcutaneous cannulation tunnel as represented by the short-dashed line. The thin arrow indicates the point of entry in the skin. (B) Longer subcutaneous cannulation tunnel as represented by the longer-dashed line, with the thin arrow indicating an entry point in the skin further from the vessel wall. The rectangles identify the positioning of the needle tip inside the arteriovenous fistula lumen. Copyright/License: Figure adapted from Servier Medical Art [22], licensed under CC BY 4.0, and from DepositPhotos [23], after purchase of a standard license that allows the use of vector illustrations on websites and for unlimited electronic display. The added designs were created by the main author of this article.

We refer to short cannulation tunnels when the needle enters the subcutaneous tissue immediately adjacent to the AVF wall, allowing the tip of the needle to be placed closer to the posterior wall of the vessel, producing a higher stream jet against the back wall (Figure 1). This type of placement is known to increase WSS and the risk of VCSS [5,14], as has already been described in a previous section. Furthermore, in AVF with dilated segments, we believe the bevel of the needle would be positioned in the transition between a higher caliber lumen (the dilated segment) and a lower caliber area (immediately after/proximal to the dilated segment). Such placement of the needle tip would cause even more turbulent blood flow from the high needle jet stream, increasing the risk of endothelium injury and restenosis.

Our study has several caveats regarding its design and sampling. First, being a single-center study, it may lack external validity. On the other hand, stenosis identification and classification were all performed by the same interventional nephrologist, which not only enhances its internal validity but also strengthens the accuracy of its findings. Second, we incurred a selection bias since there was no randomization of the sample, and the data were collected from a limited time interval (January to December 2022), which may have underestimated the number of reinterventions (both prior and subsequent to our timestamp for any given individual and/or AVF). We believe that a randomized selection with a fixed follow-up time period for each individual in a prospective study would surpass this limitation. Finally, although our center receives patients with VA complications, we do not have access to the data of the whole pool of patients from the outpatient HD centers that refer to us, which does not allow for determining the incidence of each type of stenosis, which may create a wrong perception of the frequency of restenosis in a dialysis center.

## Conclusions

Our results suggested that over 25% of all stenotic AVFs had VCSS and that these have a higher risk of needing at least one endovascular reintervention to maintain their patency and functionality. These data give an insight into how important the roles of blood flow, blood turbulence, and needle cannulation are in the development of stenotic lesions. Careful cannulation of the AVF, avoiding area technique and decreasing the risk of pseudoaneurysm formation, preference for plastic cannulae when possible, and adequate Qb are wise measures to decrease VA-related morbidity. Longer subcutaneous cannulation tunnels with shallow needle angles allow for the tip of the needle to be placed in the center of the vessel, helping decrease the incidence of VCSS, although further prospective cohort studies are warranted.
